# Changes in Child Health Care, Health, and Caregiver Mental Health During the COVID-19 Pandemic in Children with Autism and Special Health Care Needs

**DOI:** 10.1007/s10995-024-04020-3

**Published:** 2024-11-22

**Authors:** Jessica E. Rast, Kaitlin H Koffer Miller, Jennifer Bromberg, Jonas Ventimiglia, Kristy A. Anderson, Lindsay L. Shea

**Affiliations:** 1 A.J. Drexel Autism Institute, Drexel University, Jessica Rast, 3020 Market St. Suite 560, Philadelphia, PA 19104 USA; 2 Florida State University College of Social Work, Tallahassee, FL USA

**Keywords:** COVID-19, Health care, Health services, Child health, Family health, Autism, Special health care needs

## Abstract

**Purpose:**

The COVID-19 pandemic and subsequent mitigation efforts impacted communities in many ways and placed immense strain on the health care system, impacting access to services. The purpose of this study was to examine changes in prevalence of child health care, child health, and caregiver and household health within children with autism and children with special health care needs (CSHCN) pre-pandemic to early pandemic years.

**Methods:**

We examined data from the National Survey of Children’s Health to examine changes in child health care, child health, and caregiver and household health for autistic children and CSHCN from 2018 to 2021.

**Results:**

About one-third of children with autism and CSHCN missed preventive checkups due to the COVID-19 pandemic and half had virtual care in 2021. Parents of children with autism had less help with care coordination in 2020 compared to previous years. In CSHCN prevalence of anxiety increased from 2018/2019 to 2021, with a concurrent increase in need for mental health care, this was not seen in children with autism. Finally, difficulty paying medical bills and for food was less common in 2020 and 2021 (compared to 2018/2019).

**Conclusions:**

The COVID-19 pandemic changed the healthcare landscape for everyone, including children with autism and CSHCN as highlighted in this study. Understanding the disruptions and how they impacted populations differently can be helpful in informing plans long-term emergency preparedness. This planning should involve disability inclusive policies, to ensure the most vulnerable groups retain health care access as needed.

## Significance

The COVID-19 pandemic impacted physical health, mental health, and access to health care for children with special health care needs. The specific impact of the pandemic on autistic children and their families is not thoroughly documented. What this study adds: Children with autism experienced disruptions to health care during the COVID-19 pandemic. As a group with great health care and service needs, disruptions could be overly impactful to health trajectories, strengthening the need for continuous, accessible care.

## Introduction

Following the emergence of the novel coronavirus disease 2019 (COVID-19) in the U.S in January 2020, community spread of COVID-19 increased and mitigation measures such as physical distancing and travel restrictions were implemented (De Bruin et al., 2020). The ensuing pandemic resulted in far reaching impacts on employment, education, health care, support services, supply chains, the economy, and policy (Aday & Aday, 2020; Blustein et al., 2020; Hoofman & Secord, 2021). The pandemic caused immense stress on the health care system, impacting access to services. In 2020, 41% of children missed a routine health visit due to the pandemic (Teasdale et al., 2022) and there was a 33% decrease in dental visits compared to 2019 (Kranz et al., 2021). In 2021, 26% of children in the U.S. missed or delayed a preventative care visit because of COVID-19 (Lebrun-Harris et al., 2022a, 2022b). Foregone care is even more detrimental to children with increased medical or other support needs, who rely on such services to maintain health, mental health, and quality of life. This includes children with special health care needs (CSHCN) and other health, mental health, or behavioral conditions.

Even as health access decreased during the pandemic, reports of certain conditions increased. Parent-reported concerns of mood changes, anxiety, and depression increased during the pandemic (Asbury et al., 2021; Lebrun-Harris et al., 2022a, 2022b). Though CSHCN were not considered separately in these studies, pre-pandemic, anxiety, feelings of hopelessness and helplessness, and social isolation were reported more often by parents of CSHCN than parents of children with no SHCN (Caicedo, 2014). The pandemic may have heightened these differences.

As with child health and health care, caregiver and household health was impacted by the pandemic. Among caregivers with CSHCN, one study found a 26% higher odds of worse mental health during than pandemic than in the years before; similar to caregivers of children with no SHCN (20%) (Pasli & Tumin, 2023). The pandemic led to disruptions to childcare (Lebrun-Harris et al., 2022a, 2022b) and increases in unemployment (Fang et al., 2022), which led to difficulty in affording necessities for the household including food and medical expenses (Pereira & Oliveira, 2020).

Much evidence for the impact of the pandemic on child health care, child health, and caregiver and household health comes from children generally or from the broad group of CSHCN. Certain groups of children who require more health care, services, and supports were more vulnerable to the impacts of the pandemic, but these impacts are less well understood. This group includes children with intellectual and developmental disabilities (e.g. intellectual disability, autism, cerebral palsy, Down syndrome), who often require more health, behavioral, and daily support needs than other children their age. During the pandemic, most children with intellectual and developmental disabilities lost access to at least one educational or health care service due to the pandemic (Jeste et al., 2020). Caregivers of children with intellectual and developmental disabilities continued to voice concern about the long-term impacts of the disruption of needed services, educational supports, and opportunities for social engagement (Neece et al., 2020). Autistic children are among the group of children with increased support needs, often having co-occurring health and mental health conditions and receiving autism-related services and supports through school or other sources (Kerns et al., 2020). There is some data emerging specifically on the impact of the pandemic on autistic children and their families, including greater report of negative impact from school closures and disruption of developmental progress than children without autism (Genova et al., 2021; Hoofman & Secord, 2021) and worsening of behavioral issues and sleeping disturbances (Dal Pai et al., 2024).

While there are studies showing the impact of the pandemic among autistic children and CSHCN, to our knowledge there are no studies that use a nationally representative sample with a focus on a broad set of outcomes on child health care, child health, and caregiver and household health. This study focuses on the impact of the pandemic specifically on autistic children because of an increased need for interaction with the healthcare system compared to children without autism, and because of a relative lack of information of the impact of the pandemic on this group. We are comparing their outcomes to the larger group of CSHCN, who also have an increased reliance on the health care system, but their pandemic experience has been better documented. We used national U.S. survey data to examine changes in prevalence of child health care, child health, and caregiver and household health within autistic children and CSHCN from 2018 to 2021. We hypothesize that households with autistic children experienced greater decreases in child health care utilization, had greater rates of health condition increases, and had greater increases in caregiver and household hardships than households without autistic children.

## Methods

This study used data from the National Survey of Children’s Health (NSCH; 2018–2021), a nationally representative, yearly cross-sectional survey of child health and well-being in the U.S. The NSCH is supported by Health Resources and Services Administration’s (HRSA) Maternal and Child Health Bureau and conducted by the U.S. Census Bureau. Sampling and surveys were consistent across years with minor changes. In 2021, questions were added to the survey regarding the impact of the COVID-19 pandemic and public health safety measures. In each year, data was collected from June through January of the following year, with the exception of 2020, where data collection began in July (July 2020–January 2021).

### Study Sample

Children with autism were identified as those with parent report of a current autism diagnosis from a health care provider (“autism or autism spectrum disorder (ASD), Asperger’s disorder or pervasive developmental disorder (PDD)”) ages 3–17 years. Throughout this manuscript, we use “children with autism” but recognize that language preferences within the autism community vary (Vivanti, 2020). We also made comparisons to CSHCN without autism. Children were identified as having a special health care need by using a five-item screener to flag children with more use or need for health care services than typical for a child of that age (Bethell et al., 2015).

### Child Sociodemographic and Household Characteristics

Child demographic characteristics included sex (male and female), race and ethnicity (Black non-Hispanic, Hispanic, other race non-Hispanic, and white non-Hispanic), and age group (3–5, 6–11, and 12–17). We also included family structure as 1) two biological or adoptive parents, 2) two parents in the household but at least one is not biological or adoptive, 3) a single parent household, and 4) other household structures including grandparents as caregivers. Primary language spoken in the home was captured as English or other. Child health insurance coverage was captured as public insurance only, private insurance only, public and private insurance, and uninsured.

### Child Health Care Outcomes

To measure health care use changes, we examined the following survey questions about health care use in the past 12 months: any health care visits, preventive care, mental health care, specialty care, emergency department visits, and dental care. To examine unmet health and mental health care needs, caregivers were asked “during the past 12 months, was there any time when this child needed health care but it was not received?” Caregivers could endorse the specific type of care not received, including medical care and mental health services which were the two unmet needs included in this study. Finally, we examined receipt of care coordination support, where caregivers were asked if in the past 12 months, “did anyone help you arrange or coordinate this child’s care among the different doctors or services that this child uses?”.

### Child Health Outcomes

We chose to explore changes in several health conditions that may have changed in prevalence because of the COVID-19 pandemic, including asthma, anxiety, depression, and diabetes. For each of these conditions, caregivers were asked if their child had ever been diagnosed with the condition, and if so, did they currently have it. Current report of the condition was used for this study.

### Caregiver and Household Health Outcomes

We included caregiver health and household health measures in this study that have been examined in other populations over the pandemic. Caregiver health was measured using caregiver-reported mental health of the survey respondent. Household health indicators were measured by caregiver food insecurity “Which of these statements best describes your household’s ability to afford the food you need during the past 12 months?” and a response of could always afford the kinds of food we should eat was used in this study. Trouble paying medical bills was assessed using the question, “during the past 12 months, did your family have problems paying for any of this child’s medical or health care bills?”.

### COVID-19 Specific Measures

Starting in 2021, the NSCH added questions about health care use related to the pandemic. We included the following in this study: “During the past 12 months, did this child miss, delay or skip any preventive check-ups because of the coronavirus pandemic?”, and “During the past 12 months, has this child had any health care visits by video or phone?”.

### Statistical Analysis

We calculated descriptive statistics for the demographic and household characteristics of children with autism and CSHCN combined from 2018 to 2021. We used logistic regression to test for univariable significant differences in characteristics between the autism and CSHCN groups.

We then estimated the percentage of autistic children and CSHCN who experienced each of the outcomes as described above. We estimated each outcome by year (2018/2019 combined, 2020, and 2021), and then calculated a percentage point difference from the combined years 2018/2019 to 2020 and to 2021 separately for children with autism and CSHCN. We tested for statistical difference in the percentage point difference for each measure using separate logistic regression models with year as the outcome and the child/caregiver/household outcome as the sole independent variable. This was repeated by year (2018/2019 versus 2020 and 2018/2019 versus 2021) and by group (autism and CSHCN). We used *p* < *0.01* as the significance cutoff to account for multiple comparisons.

We then calculated endorsement of each COVID-19 specific measure in 2021 for children with autism and CSHCN. Finally, we performed two multivariable logistic regression models to examine the association of both COVID-19 outcomes with child and household characteristics. Analysis was performed in Stata 17 and accounted for the complex sampling design of the survey (Child and Adolescent Health Measurement Initiative, 2021; The United States Census Bureau et al., 2019).

## Results

### Child and Household Characteristics

Across all four years of the survey, there were 4,136 autistic children included in the sample and 29,592 CSHCN without autism (non-weighted). Characteristics of children with autism and CSHCN from 2018 to 2021 combined are presented in Table 1. More children with autism were male (78.4%) than CSHCN (54.6%) and were more often Hispanic (26.7% versus 21.5%). Half of children with autism were white non-Hispanic. Children with autism more often had only public insurance (40.9%) or public insurance in conjunction with private insurance (13.5%) compared to CSHCN (36.5% and 6.9%).

**Table 1:** Distribution of characteristics of autistic children and CSHCN ages 3–17, combined NSCH 2018–2021

	*Autism (n* = *4136)*		*CYSHCN (n* = *29,592)*	
	*%*	*(95% CI)*	*%*	*(95% CI)*
Male	78.4	(75.7, 80.9)	54.6**	(53.4, 55.8)
Race and ethnicity				
Black non-Hispanic	15.0	(12.7, 17.6)	16.1	(15.1, 17.0)
Hispanic	26.7	(23.1, 30.8)	21.5*	(20.2, 22.8)
Other non-Hispanic	8.8	(7.5, 10.3)	9.2	(8.6, 9.9)
White non-Hispanic	49.5	(46.1, 52.8)	53.2	(52.0, 54.5)
Age Group				
3–5	14.5	(12.4, 17.0)	11.7	(10.9, 12.5)
6–11	39.6	(36.3, 42.9)	39.6	(38.4, 40.8)
12–17	45.9	(42.5, 49.3)	48.7	(47.5, 49.9)
Family Structure				
Two biological/adoptive parents	57.6	(54.3, 60.9)	55.3	(54.0, 56.5)
Two parents, at least 1 not biological/adoptive	7.1	(5.7, 8.7)	9.1	(8.4, 9.8)
Single parent	27.7	(24.8, 30.8)	27.9	(26.8, 29.1)
Other	7.6	(6.2, 9.3)	7.7	(7.1, 8.3)
Primary Language at Home English	90.4	(87.6, 92.6)	92.5	(91.5, 93.3)
Insurance				
Public Only	40.9	(37.5, 44.4)	36.5	(35.3, 37.8)
Private Only	42.0	(38.8, 45.3)	52.3**	(51.1, 53.6)
Public and Private	13.5	(11.1, 16.3)	6.9**	(6.3, 7.6)
Uninsured	3.6	(2.6, 4.8)	4.3	(3.7, 4.9)

*p < 0.01; **p < *0.001* comparing CSHCN to children with autism

### Child Health Care Outcomes

Table 2 displays the prevalence of child health care outcomes in 2018/2019, 2020, and 2021, and the percentage point difference between 2018/2019 to 2020 and 2018/2019 to 2021. There were decreases in receipt of dental care for CSHCN (− 5.4%) from 2018/2019 to 2020 and − 3.8% from 2018/2019 to 2021. Similar but not statistically significant decreases were seen in autistic children. There were also decreases in emergency department visits for CSHCN in 2020 (− 6.2%) and 2021 (− 8.8%).

**Table 2:**
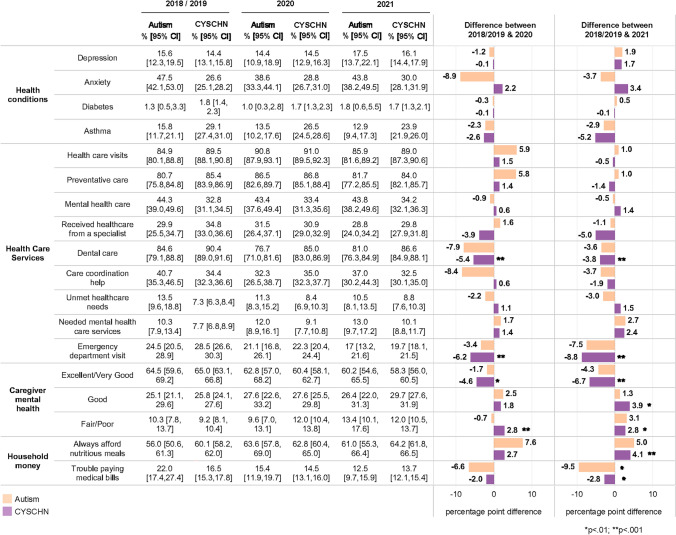
Child health care, child health, and caregiver and household measures 2018–2021

### Child Health Outcomes

Table 2 also displays prevalence of child health outcomes in 2018/2019, 2020, and 2021. The most common of the included health conditions for children with autism was anxiety, where 47.5% of parents reported their child had an anxiety diagnosis in 2018/2019. Anxiety was less commonly reported among children with autism in 2020 than in 2018/2019 (− 8.9%), but the difference was not statistically significantly different. In CSHCN, anxiety was less commonly reported overall (26.6% in 2018/2019).

### Caregiver and Household Health Outcomes

Finally, Table 2 displays caregiver health and household health outcomes in 2018/2019, 2020, and 2021. There were no statistically significant differences in caregiver health or mental health in children with autism, but for caregivers of CSHCN, there was a 4.6% point decrease in excellent/very good mental health from 2018/2019 to 2020, and a 6.7% point decrease from 2018/2019 to 2021. There were decreases among parents of children with autism (− 9.5%) and CSHCN (− 2.8%) who reported trouble paying medical bills from 2018/2019 to 2021.

### COVID-19 Specific Measures

**Fig. 1:**
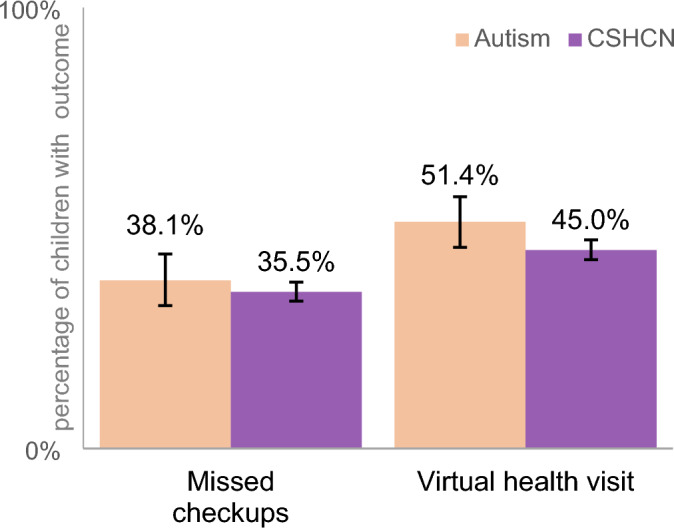
Displays the percentage of children with autism and children with special health care needs (CSHCN) who experienced each outcome in 2021 related to the pandemic

Figure 1 displays estimates on COVID-19 changes to health care in 2021. Over one-third of children with autism (38.1%) and CSHCN (35.5%) had a missed preventive check-up due to COVID in the past 12 months. About half of children with autism had a virtual health care visit in the past 12 months (51.4%).

For children with autism, most child and household characteristics were not associated with COVID-19 changes in health care in 2021 in multivariable logistic regression models (Table 3). The main exception was that worse child health was associated with increased odds of missing check-ups. This was also true in CSHCN. Family structure was also associated with missed checkups, where children in households with two parents where one was not biological or adoptive (a step or other type of parent) were less likely to have missed a checkup (autism aOR 0.48, 95% CI 0.2, 1.0; CSHCN aOR 0.72, 95% CI 0.5, 1.0) than children in a home with two biological or adoptive parents. In CSHCN, age was associated with virtual care, where CSHCN ages 3–5 years had lower odds of virtual care than CSHCN ages 12–17 (aOR 0.65, 95% CI 0.5, 0.9).

**Table 3:** Associations of child and family characteristics with COVID-19 changes in 2021

	Missed check-ups		Virtual care	
	Autism	CYSHCN	Autism	CYSHCN
	aOR (95% CI)	aOR (95% CI)	aOR (95% CI)	aOR (95% CI)
Child characteristics				
Female	0.77 (0.4, 1.3)	0.90 (0.7, 1.1)	1.56 (0.9, 2.6)	1.11 (0.9, 1.3)
Race/ethnicity				
Black non-Hispanic	0.72 (0.4, 1.4)	1.23 (0.9, 1.6)	0.49 (0.2, 1.1)	0.74 (0.5, 1.0)
Hispanic	1.16 (0.6, 2.3)	1.05 (0.8, 1.4)	1.63 (0.9, 3.0)	0.82 (0.6, 1.1)
Other non-Hispanic	1.06 (0.5, 2.1)	1.39 (1.0, 1.9)	1.00 (0.5, 1.9)	0.87 (0.7, 1.2)
White non-Hispanic	1.0		1.0	
Age				
3–5	0.55 (0.3, 1.1)	0.92 (0.7, 1.2)	1.07 (0.6, 1.9)	0.65 (0.5, 0.9)
6–11	0.77 (0.5, 1.3)	1.03 (0.8, 1.3)	0.70 (0.4, 1.1)	0.82 (0.7, 1.0)
12–17	1.0		1.0	
Child health				
Very good	1.0		1.0	
Good	2.34 (1.3, 4.1)	1.37 (1.1, 1.7)	1.26 (0.7, 2.2)	1.30 (1.0, 1.6)
Fair / poor	2.34 (1.0, 5.7)	2.01 (1.1, 3.5)	3.43 (1.2, 9.6)	1.86 (1.1, 3.2)
Insurance				
Public Only	0.99 (0.6, 1.7)	0.97 (0.8, 1.2)	1.34 (0.8, 2.2)	0.85 (0.7, 1.1)
Private Only	1.0		1.0	
Public and Private	0.64 (0.3, 1.3)	0.90 (0.6, 1.3)	1.71 (0.9, 3.4)	1.60 (1.1, 2.4)
Family characteristics				
Family Structure				
Biological/adoptive parents	1.0		1.0	
Two parents, not biological	0.48 (0.2, 1.0)	0.72 (0.5, 1.0)	0.61 (0.3, 1.3)	0.69 (0.5, 0.9)
Single parent / other	0.70 (0.4, 1.2)	0.88 (0.7, 1.1)	0.98 (0.6, 1.6)	0.92 (0.7, 1.1)
Non-English language	0.63 (0.2, 2.0)	0.71 (0.4, 1.2)	0.66 (0.2, 2.1)	1.18 (0.7, 1.9)

## Discussion

The COVID-19 pandemic changed the healthcare landscape for everyone, including children with autism and CSHCN as highlighted in this study. Health care appointments were missed, caregiver mental health worsened, and virtual visits were common. In the pandemic years, especially 2021, there were decreases in emergency department care for autistic children and CSHCN. This has been seen in other research, where decreases are thought to be caused by a combination of decreases in contraction of infectious disease and injury, and by caregivers forgoing necessary care to avoid the hospital for serious conditions including appendicitis (Pines et al., 2021).

Beyond health care services, this study examined the parent-reported prevalence of certain conditions that have been found to increase over the pandemic population in other studies and populations. We did not find statistically significant changes in depression, anxiety, diabetes, or asthma for autistic children or CSHCN from pre-pandemic to 2020 or 2021 at the *p* < *0.01* level. However, some larger but not statistically significant differences are important to discuss, as they have been found in previous studies. Our study found a 3.4% point increase in anxiety from 2018/2019 to 2021 in CSHCN. Prior studies have shown increased rates of anxiety symptoms in children during this time (Racine et al., 2021), though our study examined an anxiety diagnosis, not anxiety symptoms. Conversely, we found a large decrease in anxiety reported in 2020 compared to 2018/2019 for autistic children. Emerging qualitative research with autistic adults reported improved mental health, including anxiety and depression, during the COVID-19 pandemic (Bundy et al., 2022; Realpe et al., 2023). However, diagnosis of a new condition during this time period would have been difficult, as there was a marked decrease in care receipt and access. Parents of children with autism had less help with care coordination in 2020, while CSHCN experienced no change, suggesting a lack of connection to services that may be needed for diagnosis. Previous research found that parents of CSHCN reported that the pandemic amplified their difficulty accessing and coordinating care for their children (Cohen et al., 2022; Currie et al., 2023). If this is also true in parents of autistic children, it could explain a reduction in anxiety diagnosis during the pandemic as a lack of connection to providers who could make such a diagnosis. For children who need multiple services, specialties, and providers, care coordination is important in reducing caregiver burden and making care more accessible (Boudreau et al., 2014). With office closures and the reduction in overall visits, care coordination was more difficult to come by, but it remains an important part of care provision for children with complex needs.

When examining household health, we found decreases in parent-reported difficulty paying medical bills and an increase in the ability to afford nutritious meals in both 2020 and 2021 (compared to 2018/19). Several policies initiated during the COVID-19 pandemic may have led to these financial improvements. Care for COVID-19, including vaccinations and testing, was largely free. Health insurance coverage was also expanded to allow more families to access Medicaid coverage during this time. The federal government also made efforts to facilitate access to food, including expanding access to and payment from safety net programs, and schools made free lunches available to students. One-time stimulus payments were useful in offsetting costs (Garner et al., 2020). Our findings suggest these efforts had a positive financial impact on families of children with autism and CSHCN.

The long-lasting impact of these changes is yet to be quantified, but understanding the disruptions and how they impacted populations differently can be helpful in informing plans for system improvement.

### Child and Family Characteristics Associated with COVID-19 Impacts

We did not find many child or family characteristics that were associated with missed check-ups or use of virtual care for children with autism or CSHCN. One consistent finding was that children with autism and CSHCN with poorer health had greater odds of missed check-ups and of virtual care than children with excellent health. Children with poorer health have greater need of interaction with the health care system which likely explains an increase in missed visits, as more visits are scheduled and could therefore be missed. A pandemic study of children with medical complexity found that many specialist visits were missed during the pandemic (Baumbusch et al., 2022). Virtual visits likely increased in this population for the same reason, in additional to avoiding in-person visits to reduce risk of COVID-19 exposure (Malhotra et al., 2021; Teasdale et al., 2022). With a quick implementation of virtual visits nationwide, there has been inconsistency in the quality, accessibility, and efficacy of virtual care (Garfan et al., 2021) and nearly half (44%) of parents of children with disabilities reported low satisfaction with virtual services during the pandemic (Murphy et al., 2021). Virtual care is an important modality for planning for health care provision during emergencies, but accessibility and quality for a variety of populations should be addressed.

Family structure was the other consistent association with missed visits and virtual care. Families with two parents where one was not adoptive or biological (referred to hereafter as stepfamilies) had decreased odds of missing a check-up compared children in households with two biological/adoptive parents, for both autistic children and CSHCN. There is limited research about stepfamily experiences during the pandemic. Research on child health pre-pandemic has found that stepfamilies are larger and have more financial barriers to health care, leading to an increase in unmet care needs compared to families with two biological/adoptive parents (Irvin et al., 2018). During the pandemic, families were isolated and the responsibility of caring for children was removed from the larger community and placed within the immediate family (Power, 2020). A child living in a stepfamily often has a second household with which care and medical responsibilities can be shared, reducing some barriers to care during the pandemic. Perhaps conversely, we also found reduced odds of virtual care for children in stepfamilies. Studies in children with neurodevelopmental disorders have found that single parent families face barriers to accessible virtual care including multitasking during appointments and caring for multiple children (Hosley, 2022). Larger family size in stepfamilies, and the larger role of childcare responsibility by the biological parent in the household, could make virtual care more difficult for stepfamilies. Family composition should be considered in future planning.

### Limitations

This study has several limitations. First, this study uses repeated cross sections (each year of the survey is a new sample) and is not able to assess causality of the effect of the pandemic on outcomes. Second, the sampling timeframe and retrospective questions may not have captured the full pandemic experience. The 2020 survey was administered from July 2020 to January 2021, and caregivers were often asked to recall events within the past 12 months, which would have fallen before the start of the pandemic (March 2020). To account for this, we included the 2021 survey, which was administered from June 2021 to January 2022. However, the 2021 survey would not capture events that occurred before June 2020, which would miss events at the beginning of the pandemic. Third, this survey relies on caregiver report of events, which must be recalled over a long period which was very tumultuous. Finally, smaller sample size in children with autism may have obscured some meaningful differences.

## Conclusions

This study examined child health care, child health, and caregiver and household health in children with autism and CSHCN before and during the pandemic. We found decreases in access to some types of care, such as dental care, emergency department visits, and care coordination, for autistic children and CSHCN. Commensurate increases in virtual care were a promising solution to these disruptions. Virtual platforms should ensure they are accessible to all patients and provide high quality services. Findings strengthen arguments that we should prepare for future service system interruptions to mitigate such disruptions to care. This emergency preparedness planning should involve disability inclusive policies, to ensure the most vulnerable groups retain health care access as needed. It is crucial to provide consistent and appropriate health care to ensure the health and wellbeing of vulnerable populations and improve outcomes both directly and indirectly related to health.

## Data Availability

Data can be accessed through the Data Resource Center for Child and Adolescent Health at http://childhealthdata.org/
